# A combination of silver nanoparticles and visible blue light enhances the antibacterial efficacy of ineffective antibiotics against methicillin-resistant *Staphylococcus aureus* (MRSA)

**DOI:** 10.1186/s12941-016-0164-y

**Published:** 2016-08-17

**Authors:** Fatma Elzahraa Akram, Tarek El-Tayeb, Khaled Abou-Aisha, Mohamed El-Azizi

**Affiliations:** 1 Department of Microbiology, Immunology, and Biotechnology, German University in Cairo, GUC, New Cairo City, Cairo Egypt; 2 National Institute for Laser Enhanced Sciences, Cairo University, Cairo, Egypt

**Keywords:** Nonconventional antimicrobials, Double and triple combinations, Multidrug-resistance, Checkerboard assay, Linezolid, Vancomycin, Azithromycin, Clarithromycin

## Abstract

**Background:**

Silver nanoparticles (AgNPs) are potential antimicrobials agents, which can be considered as an alternative to antibiotics for the treatment of infections caused by multi-drug resistant bacteria. The antimicrobial effects of double and triple combinations of AgNPs, visible blue light, and the conventional antibiotics amoxicillin, azithromycin, clarithromycin, linezolid, and vancomycin, against ten clinical isolates of methicillin-resistant *Staphylococcus aureus* (MRSA) were investigated.

**Methods:**

The antimicrobial activity of AgNPs, applied in combination with blue light, against selected isolates of MRSA was investigated at 1/2–1/128 of its minimal inhibitory concentration (MIC) in 24-well plates. The wells were exposed to blue light source at 460 nm and 250 mW for 1 h using a photon emitting diode. Samples were taken at different time intervals, and viable bacterial counts were determined. The double combinations of AgNPs and each of the antibiotics were assessed by the checkerboard method. The killing assay was used to test possible synergistic effects when blue light was further combined to AgNPs and each antibiotic at a time against selected isolates of MRSA.

**Results:**

The bactericidal activity of AgNPs, at sub-MIC, and blue light was significantly (p < 0.001) enhanced when both agents were applied in combination compared to each agent alone. Similarly, synergistic interactions were observed when AgNPs were combined with amoxicillin, azithromycin, clarithromycin or linezolid in 30–40 % of the double combinations with no observed antagonistic interaction against the tested isolates. Combination of the AgNPs with vancomycin did not result in enhanced killing against all isolates tested. The antimicrobial activity against MRSA isolates was significantly enhanced in triple combinations of AgNPs, blue light and antibiotic, compared to treatments involving one or two agents. The bactericidal activities were highest when azithromycin or clarithromycin was included in the triple therapy compared to the other antibiotics tested.

**Conclusions:**

A new strategy can be used to combat serious infections caused by MRSA by combining AgNPs, blue light, and antibiotics. This triple therapy may include antibiotics, which have been proven to be ineffective against MRSA. The suggested approach would be useful to face the fast-growing drug-resistance with the slow development of new antimicrobial agents, and to preserve last resort antibiotics such as vancomycin.

## Background

Treatment of infections caused by *Staphylococcus aureus* has become more difficult because of the emergence of multidrug-resistant isolates [1, 2]. Methicillin-resistant *S. aureus* (MRSA) presents problems for patients and healthcare facility-staff whose immune system is compromised, or who have open access to their bodies via wounds, catheters or drips. The infection spectrum ranges from superficial skin infections to more serious diseases such as bronchopneumonia [3].

Failure of antibiotics to manage infections caused by multidrug-resistant (MDR) pathogens, especially MRSA, has triggered much research effort for finding alternative antimicrobial approaches with higher efficiency and less resistance developed by the microorganisms. Silver has long been known to exhibit antimicrobial activity against wide range of microorganisms and has demonstrated considerable effectiveness in bactericidal applications [4] and silver nanoparticles (AgNPs) have been reconsidered as a potential alternative to conventional antimicrobial agents [5].

It has been estimated that 320 tons of nanosilver are used annually [6] with 30 % of all currently registered nano-products contain nanosilver [7]. The use of AgNPs alone or in combination with other antimicrobial agents has been suggested as a potential alternative for traditional treatment of infections caused by MDR pathogens [5]. AgNPs were found to exhibit antibacterial activity against MRSA in vitro when tested alone or in combination with other antimicrobial agents [8–10].

Metal nanostructures attract a lot of attention due to their unique properties. AgNPs is a potential biocide that has been reported to be less toxic compared to Silver ions [11]. AgNPs can be incorporated into antimicrobial applications such as bandages, surface coatings, medical equipment, food packaging, functional clothes and cosmetics [12].

Blue light is recently attracting increasing attention as a novel phototherapy-based antimicrobial agent that has significant antimicrobial activity against a broad range of bacterial and fungal pathogens with less chance to resistance development compared to antibiotics [13, 14]. Further, blue light has been shown to be highly effective against MRSA and other common nosocomial bacterial pathogens [15, 16].

The present investigation aims to evaluate the effectiveness of triple combination of AgNPs, blue light and the conventional antibiotics vancomycin, linezolid, amoxicillin, azithromycin, and clarithromycin against clinical isolates of MRSA. To the best of our knowledge, this is the first study, which utilizes this triple combination against pathogenic bacteria.

## Methods

### Chemicals

Unless otherwise indicated all chemicals were purchased from Sigma-Aldrich, USA.

### Antibiotics

Amoxicillin (AMX), oxacillin (OXA), vancomycin (VAN) were purchased from Sigma Chemical Co., ST. Louis, Missouri, USA. Linezolid (LNZ) was provided by Pharmacia & Upjohn, Kalamazoo, MI, USA. Azithromycin (AZM) was provided by Pfizer, USA. Clarithromycin (CLR) was provided by Abbott Laboratories, USA.

### Microorganisms

Ten clinical MRSA isolates were collected from The National Cancer Institute and from Abbasseya Hospital in Cairo, Egypt. The collected isolates were identified using conventional microbiological techniques.

According to genotyping results, the isolates were sub-classified into 14 different pulsed field patterns, 11 *spa*-types and 8 *multiple locus sequence typing* (MLST) sequence types. The pulsed field type A was the predominant pulsed field type, which corresponded to *spa*-type t-037 and MLST sequence type ST-239, and belonged to clonal complex 8 (CC8) according to eBURST analysis (Table 1) (Unpublished data, Master Thesis, Moussa et al. 2010).

**Table 1:** Characteristics of the MRSA clinical isolates used in this study

Isolate designation	Spa-repeats	MLST	Clonal complex (eBURST)	*SCCmec*
C51	t-186	ST-88	CC88	IIIa
C6	t-5711	ST-22	CC22	IVa
C43	t-037	ST-239	CC8	III
N11	t-363	ST-239	CC8	III
N5	t-037	ST-239	CC8	III
N8	t-037	ST-239	CC8	III
C34	t-037	ST-239	CC8	III
C19	t-037	ST-239	CC8	III
C12	t-037	ST-239	CC8	III
C41	t-1234	ST-97	CC97	III

### Oxacillin susceptibility

The isolates were inoculated onto Mueller–Hinton agar (Lab M, UK) plates supplemented with 4 % NaCl and 6 µg/mL oxacillin, followed by incubation at 37 °C for 24 h. Isolates that showed more than one colony were considered as MRSA [17].

### Preparation of the AgNPs

The AgNPs used for the purpose of this research are silver magnetite nanoparticles. To prepare the AgNPs, 0.127 g silver nitrate were dissolved in 75 mL of distilled water then 10 mL of an aqueous solution containing 0.08 g trisodium citrate and 0.2 g polyvinylpyrrolidone (PVP) were added. Ten milliliter of 0.1 M sodium borohydride were dissolved and added to the mixture. The solution turned dark brown indicating the conversion of silver nitrate to silver nanoparticles. The nanoparticles were characterized spectrophotometrically, where a surface plasmon resonance peak appeared between 390 and 410 nm [18]. The particles size was also characterized by Malvern Zetasizer Nano ZS (United Kingdom) and by Tecnai G20, Super twin, double tilt (FEI) ultra-high resolution Transmission Electron Microscope, which showed a uniform distribution of the nanoparticles, with an average size of 15–20 nm.

### Susceptibility of the isolates to AgNPs and the antibiotics

MIC of the AgNPs was determined by the broth microdilution method using cation-adjusted Mueller–Hinton broth (MHB) based on the guidelines of the Clinical Laboratory Standard Institute (CLSI) [19]. The minimum bactericidal concentration (MBC) was determined by streaking 10 µL samples from bacterial cultures supplemented with AgNPs or the antibiotics at their MICs and higher concentrations, onto the surfaces of Muller Hinton agar plates. After a 24 h incubation period, the number of colony forming units per mL (CFU/mL) was determined and the MBC, defined as the concentration that kills 99.9 % of bacteria, was recorded.

### Double combination of AgNPs with blue light against MRSA

AgNPs were tested at 1/2, 1/4, 1/8, 1/16, 1/32, 1/64 and 1/128 of its MIC in 24-wells plates. Briefly, bacterial suspensions were pipetted into the wells, which contained the AgNPs at the tested concentrations in MHB to give an initial inoculum size of 1 × 10^5^ CFU/mL and a final volume of 2 mL/well. The wells were exposed to visible blue light source at 460 nm and 250 mW for 1 h using Photon Emitting Diode (Photon Scientific, Egypt). Samples were taken after 0, 2, 4, 6, 8 and 10 h of inoculation, where viable bacterial counts were determined. Briefly, 10 µL aliquots were withdrawn and spread onto nutrient agar plates before being incubated at 37 °C for 24 h. The same procedure was repeated with nanoparticles-free and light-free wells. The experiment was performed in triplicates and the results were compared to drug-free samples.

### Double combination of AgNPs with the antibiotics against MRSA

The efficiency of double combination of AgNPs and amoxicillin, vancomycin, linezolid, azithromycin, or clarithromycin against the ten clinical isolates of MRSA was assessed by the checkerboard method. The combination response was evaluated by calculation of the Fraction Inhibitory Index (∑ FIC) as follow:

$$\sum {\text{ FIC}} = \frac{ \, MIC \, of \, drug \, A, \, in \, combination}{MIC \, of \, drugA, \, tested \, along} \, + \, \frac{MIC \, of \, drug \, B, \, in \, combination}{MIC \, of \, drug \, B, \, tested \, along}$$

The interaction is defined as synergistic if the FIC index is 0.5 or less; indifferent, if the FIC index is >0.5 and <4; and antagonistic if the FIC index is >4 [20].

### Triple combination of AgNPs, blue light, and the antibiotics against MRSA

The purpose of this experiment was to test the effectiveness of AgNPs in combination with blue light and each of the following antibiotics at a time: amoxicillin, vancomycin, linezolid, azithromycin, or clarithromycin, against selected isolates of MRSA. Two isolates from the combination of AgNPs and each of the tested antibiotics were chosen on the basis of the synergistic response in the checkerboard assay.

The experiments were carried out in 24 multi-well plates where eight wells were designated as: drug- and light-free, blue light exposure, AgNPs alone, the antibiotic alone, blue light and AgNPs, blue light and the antibiotic, AgNPs and the antibiotic, and finally, the triple combination blue light, AgNPs and the antibiotic. The 24 multi-well plates were used because the diameter of their wells fits the tip of the Photon Emitting Diode, where the diode was placed at a distance of 5 mm over the surface of the bacterial culture in the well to ensure optimal exposure to the light and reduce light scattering. Only the wells in the four corners of one plate were used in parallel treatments to avoid the scattered light from adjacent wells, if any; all other wells were left empty.

The AgNPs and the antibiotics were tested at concentrations that resulted in the best combination in checkerboard assay against the selected isolates. Bacterial suspensions were pipetted into the wells, which contained the AgNPs alone or in combination with the antibiotics at the test concentrations in MHB to give an initial inoculum size of 1 × 10^5^ CFU/mL and a final volume of 2 mL/well. The wells designated for light treatment were exposed to the light source emitting blue light at a wavelength of 460 nm for 1 h. The plates were then incubated at 37 °C for 24 h after which viable cell counts were determined. The experiment was performed in triplicate, and the results obtained were compared to the drug- and blue light-free wells.

### Effects of triple combination of AgNPs, blue light, and azithromycin on MRSA isolate using transmission electron microscopy (TEM)

Ten milliliter of MHB medium were inoculated with 1 × 10^5^ CFU/mL of MRSA isolate (N8) in 15 mL conical centrifuge tubes (Falcon, USA). The suspensions were then incubated at 37 °C for 4 h till the bacteria reached the logarithmic phase. The suspensions were then centrifuged at 2800×*g* for 10 min and the cell pellets were re-suspended in 10 mL of the fresh drug-free MHB, or containing 0.25 µg/mL (1/16 MIC) of AgNPs, or 0.25 µg/mL of azithromycin or both agents. Two milliliter aliquots of the suspension were transferred to 24 multi-wells plates. The plates were incubated at room temperature during which the blue light wells were exposed to the light at 460 nm for 1 h. One milliliter samples were then taken and prepared for TEM as previously described [21]. Briefly, the samples were centrifuged, and the bacterial pellets were fixed in 1 mL of 3 % glutaraldehyde for 2 h and then centrifuged and washed with 7.2 % phosphate buffer. A secondary fixative, osmium tetraoxide, was then added to the pellets, incubated for 1 h before being washed with phosphate buffer saline. The samples were then subjected to a series of dehydration steps using different concentrations of ethanol, starting with ethanol 50–95 %. During each step, the samples were left for 10 min and then put in absolute ethanol for 20 min. The samples were then embedded in resin blocks that were subsequently cut into semi- then ultra-thin thickness and finally stained with uranyl acetate and lead citrate before being examined by TEM JEOL (JEM-1400). The results were compared to drug- and light-free control experiments.

### Statistical analysis

The statistical analysis of the data was done using GraphPad Prism (version 5.0) software. One-way- and two-way analysis of variance (ANOVA) were used to test the significance among the different treatment groups, and 5 % error was accepted in the statistics. Error bars in the graphical presentation of data express the standard deviation of the means between samples.

## Results

### Susceptibility of the isolates to AgNPs and the antibiotics

The MIC of AgNPs was found to be 4 µg/mL with MBC range of 8–16 µg/mL, and MBC_90_ (The minimum bactericidal concentration of the antibiotic required to kill 99.9 % of bacteria in 90 % of the isolates) was 8 µg/mL (Table 2). Vancomycin is the only antibiotic, which showed activity against the tested isolates with MIC_90_ (the minimum inhibitory concentration of the antibiotic required to inhibit the growth of 90 % of the isolates) and MBC_90_ values of 2 and 8 µg/mL, respectively (Table 2). The isolates were resistant to linezolid with MIC_90_ of 32 µg/mL, and to amoxicillin, azithromycin and clarithromycin with MIC_90_ >64 µg/mL.

**Table 2:** Susceptibility of the tested isolates to AgNPs and the antibiotics

Antimicrobial agents	Concentration (µg/mL)^a^	
	MIC_90_	MBC_90_
AgNPs	4	8
Amoxicillin	>64	>64
Azithromycin	>64	>64
Clarithromycin	>64	>64
Linezolid	32	>64
Vancomycin	2	8

^a^MIC_90_: The minimum inhibitory concentration of the antibiotic required to inhibit the growth of 90 % of the isolates. MBC_90_: The minimum bactericidal concentration of the antibiotic required to kill 99.9 % of bacteria in 90 % of the isolates

### Combination of AgNPs with blue light against MRSA

**Fig. 1:**
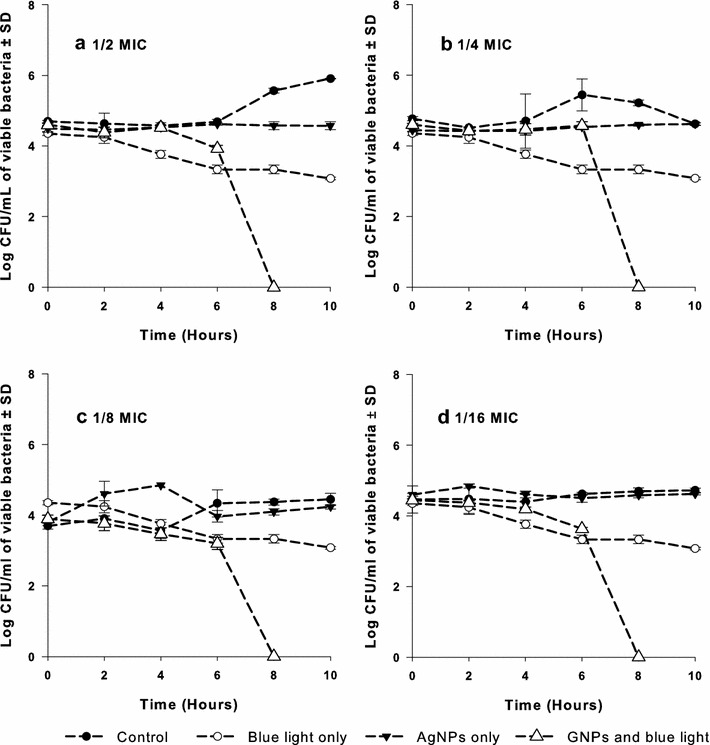
Antimicrobial activity of the AgNPs at different concentrations in combination with blue light against MRSA isolates. Cell suspensions were exposed to either the silver compound alone at sub-MICs (**a** 1/2, **b** 1/4, **c** 1/8, and **d** 1/16 MIC), or blue light alone at 460 nm and 250 mW for 1 h, or combination of both agents. Viable colony count was recorded as mean ± SD of three independent experiments. *AgNPs* silver nanoparticles, *CFU* colony forming unit, *MIC* minimum inhibitory concentration, *SD* standard deviation

The antimicrobial activity of AgNPs in combination with blue light against one of the MRSA isolates was investigated. The AgNPs were tested at 1/2, 1/4, 1/8, 1/16, 1/32, 1/64 and 1/128 of its MIC in 24-wells plates. The antimicrobial activity of these combinations against the tested isolate was significantly higher (p < 0.001) than each agent alone. All bacteria were killed after 8 h of exposure to the combined therapy at all tested concentrations. Figure 1 shows the results for the combinations tested at 1/2, 1/4, 1/8, and 1/16 of the MIC of AgNPs (data for lower concentrations are not shown).

### Combination of AgNPs with the antibiotics against MRSA

**Fig. 2:**
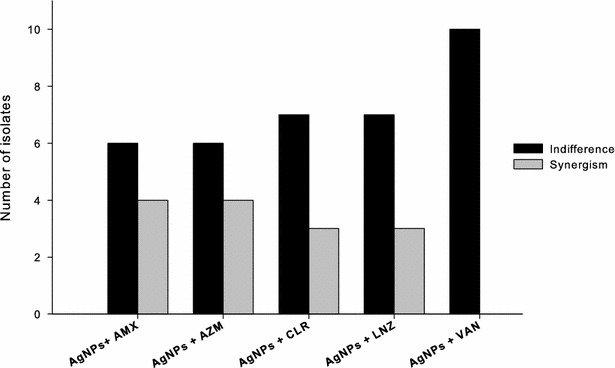
Double combination of AgNPs with the amoxicillin, vancomycin, linezolid, azithromycin or clarithromycin against ten MRSA isolates. The combination was assessed by the checkerboard method and the response was evaluated by calculation of the fraction inhibitory index (FIC) as follow: synergistic if the FIC index is 0.5 or less, indifference if the FIC index more than 0.5 and less than four, and antagonistic if the FIC index more than four. *AgNPs* silver nanoparticles, *AMX* amoxicillin, *AZM* azithromycin, *CLR* clarithromycin, *LNZ* linezolid, *VAN* vancomycin

The efficiency of the double combination of the AgNPs and each of amoxicillin, vancomycin, linezolid, azithromycin, or clarithromycin, against the ten clinical MRSA isolates was assessed using the checkerboard method. The combination of AgNPs with amoxicillin resulted in synergistic activity against four isolates whereas indifference response was observed in six isolates. Similar results were observed when the AgNPs were combined with azithromycin, clarithromycin or linezolid, where synergism was observed against 4, 3 and 3 isolates, respectively, whereas indifferent interaction prevailed for the remaining isolates. On the other hand, combination of AgNPs with vancomycin was indifferent for all tested isolates (Fig. 2).

### Triple combination of AgNPs, blue light, and the antibiotics against MRSA isolates

The effectiveness of the AgNPs in combination with blue light and amoxicillin, linezolid, azithromycin, or clarithromycin, was tested against selected isolates of MRSA. Two isolates from each combination of AgNPs and antibiotic were selected based on the synergistic results of the checkerboard assay. Vancomycin was excluded because its combination with the AgNPs was indifferent against all isolates.

**Fig. 3:**
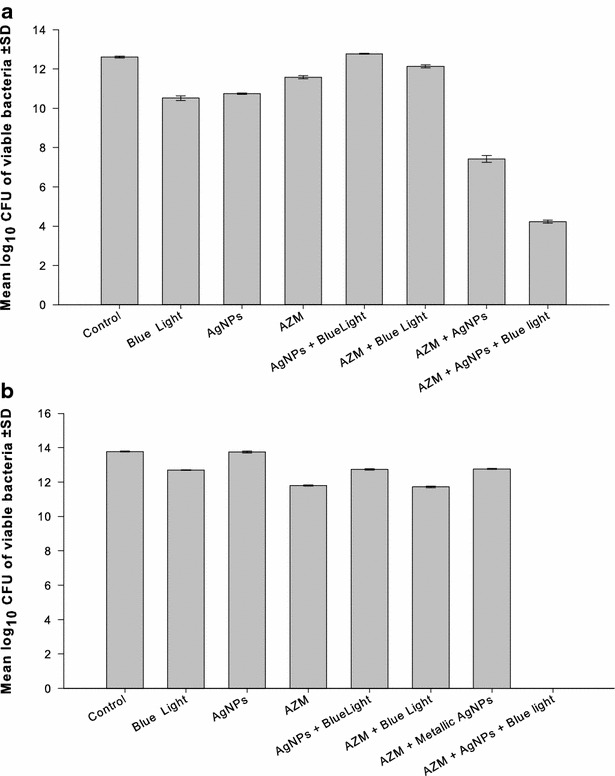
Combination of AgNPs, blue light, and azithromycin against two isolates of MRSA. The triple combination of AgNPs with the blue light and azithromycin against two isolates of MRSA was assessed. The isolates were selected on the basis of synergistic response in checkerboard assay. Based on the best result of the combination in the checkerboard assay, the concentrations of the two agents were used as follow: **a** Isolate C41: AgNPs at 1/16 of the MIC, and azithromycin at 2 µg/mL. **b** Isolate N8: AgNPs at 1/16 of the MIC, and azithromycin at 0.25 µg/mL. *AgNPs* silver nanoparticles, *CFU* colony forming unit, *AZM* azithromycin, *SD* standard deviation

The AgNPs and the antibiotics were tested at the concentrations, which gave the best results in checkerboard assay. Isolates N8 and C41 were used to assess the triple combination of AgNPs at 1/16 MIC, the blue light, and azithromycin at 0.25 and 2 µg/mL, respectively. The triple combination resulted in significantly higher (p < 0.001) killing effect of isolate C41 with log_10_ CFU/mL reductions of 8.4, 3.2, compared to the drug-free samples and to the double combinations of the antibiotic with the AgNPs (Fig. 3a). The triple combinations against isolate N8 resulted in killing of all bacteria compared to all other treatments, which showed lower activity with log_10_ CFU/mL reduction range of 1.0–2.0 (Fig. 3b).

**Fig. 4:**
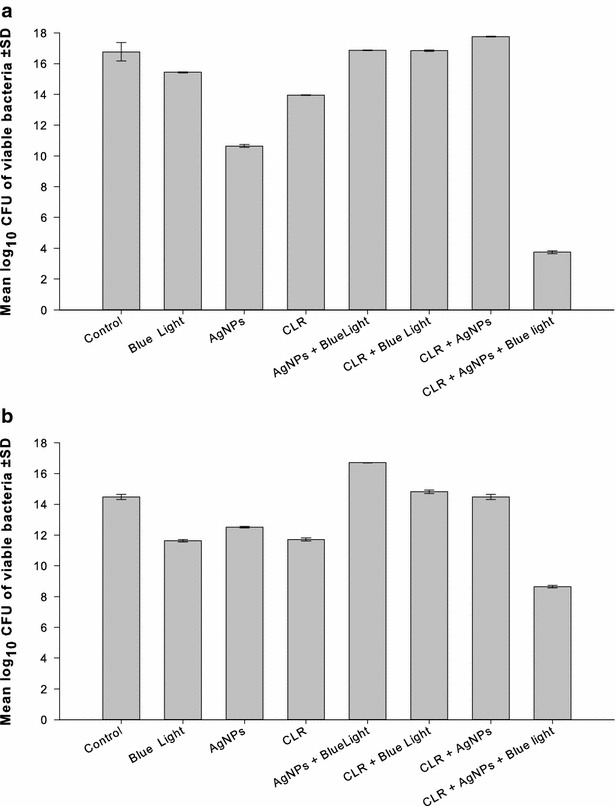
Combination of AgNPs, blue light, and clarithromycin against two isolates of MRSA. The triple combination of AgNPs with the blue light and clarithromycin against two isolates of MRSA was assessed. The isolates were selected on the basis of synergistic response in checkerboard assay. Based on the best result of the combination in the checkerboard assay, the concentrations of the two agents were used as follow: **a** Isolate C51: AgNPs at 1/8 of the MIC, and azithromycin at 0.25 µg/mL. **b** Isolate C41: AgNPs at 1/512 of the MIC, and azithromycin at 0.25 µg/mL. *AgNPs* silver nanoparticles, *CFU* colony forming unit, *CLR* clarithromycin, *SD* standard deviation

Triple combinations that included clarithromycin were tested against isolates C51 and C41, at 1/8 and 1/512 of the MIC, respectively, of the AgNPs and 0.25 µg/mL of the antibiotic. The bactericidal activity of the three-agent combination was significantly higher (p < 0.001) than that attained with other treatment combinations with log_10_ CFU/mL reduction of 13.02 and 5.84 compared to the control of the two isolates, respectively (Fig. 4a, b).

**Fig. 5:**
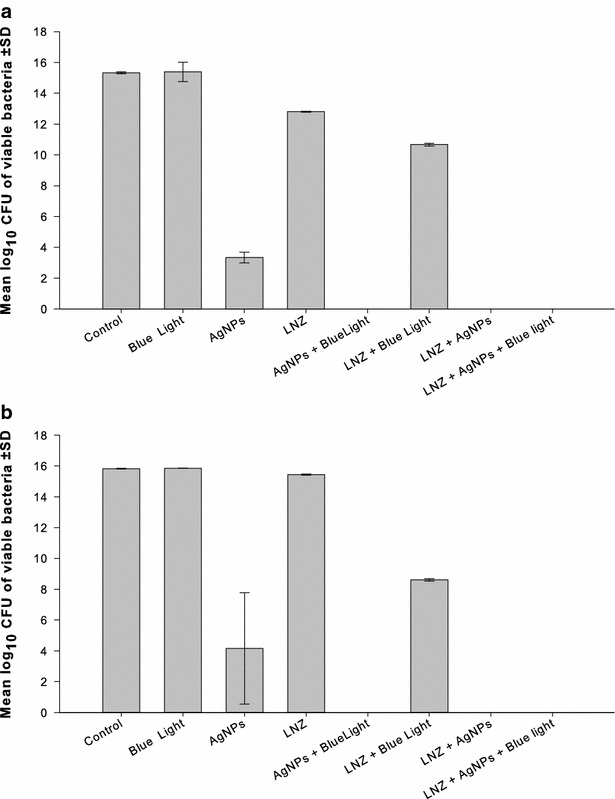
Combination of AgNPs, blue light, and linezolid against two isolates of MRSA. The triple combination of AgNPs with the blue light and linezolid against two isolates of MRSA was assessed. The isolates were selected on the basis of synergistic response in checkerboard assay. Based on the best result of the combination in the checkerboard assay, the concentrations of the two agents were used as follow: **a** Isolate C19: AgNPs at 1/2 of the MIC, and azithromycin at 0.25 µg/mL. **b** Isolate N5: AgNPs at 1/2 of the MIC, and azithromycin at 8 µg/mL. *AgNPs* silver nanoparticles, *CFU* colony forming unit, *LNZ* linezolid, *SD* standard deviation

The antimicrobial efficacy of linezolid at 0.25 and 8 µg/mL was evaluated against isolate C19 and N5, respectively, when combined with the silver compound at its 1/2 MIC and blue light. Synergistic interaction was observed when the AgNPs were combined with either the antibiotic or the blue light or with both of them, where the bacteria were completely killed following treatment with all combinations (Fig. 5a, b). The same effect was observed when amoxicillin at 1 and 0.25 µg/mL was combined with blue light and AgNPs at 1/32 and 1/256 of its MIC against isolates C12 and N8, respectively (data not shown).

### TEM examination of MRSA isolate (N8) after treatment with the AgNPs, blue light, and azithromycin alone or in triple combination

**Fig. 6:**
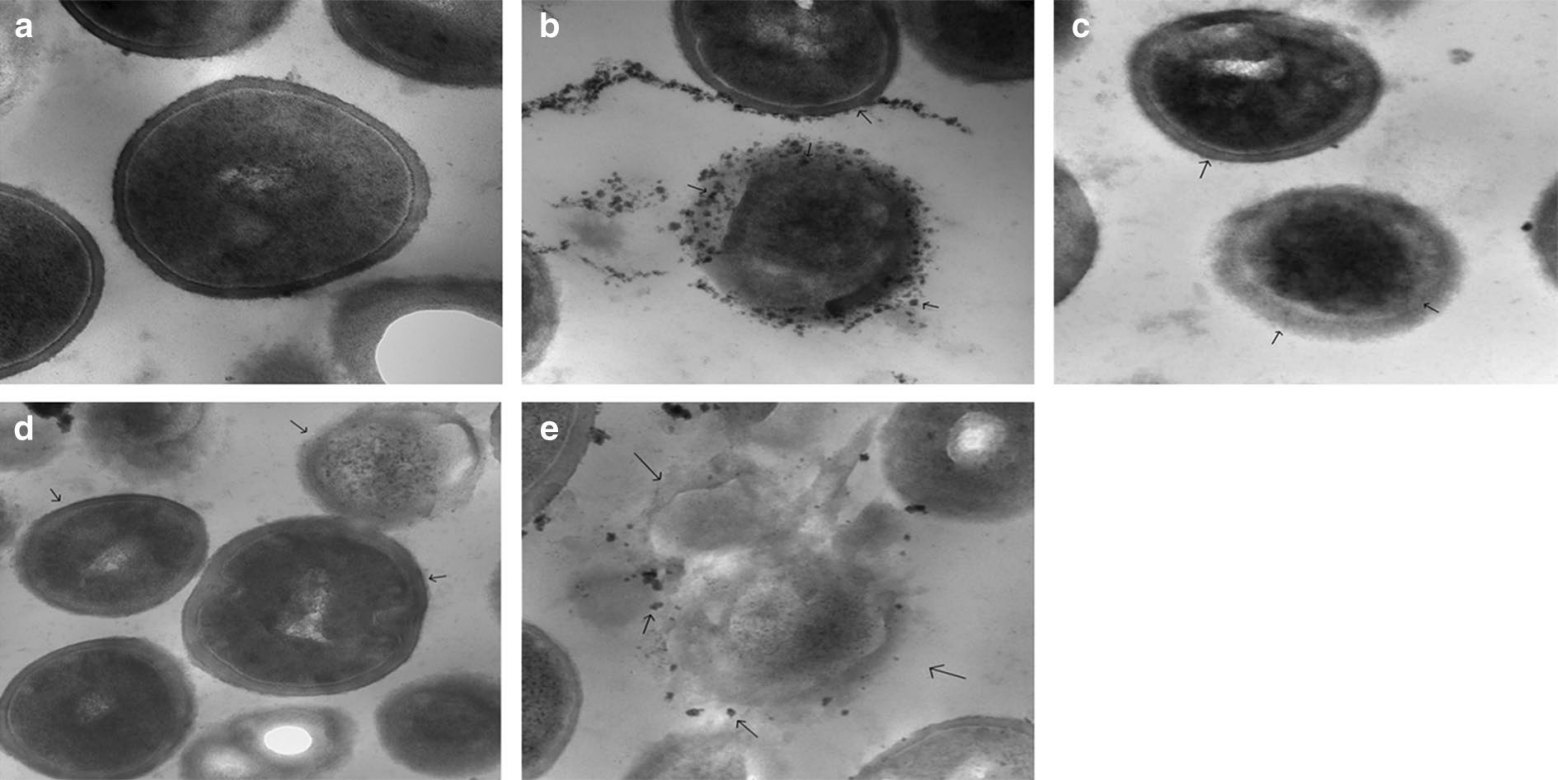
Visualization of the effect of combination of AgNPs, blue light, and azithromycin on MRSA isolate N8 using transmission electron microscope (TEM). The antimicrobial efficacy of the AgNPs at 1/16 MIC, blue light and azithromycin at 0.25 µg/mL alone and in triple combination against isolate N8 was visualized by TEM at ×80,000. The photos show the response of the bacteria to the following treatments: **a** Drug-free and light-free (control). **b** AgNPs alone at 1/16 of its MIC. **c** Blue light exposure at 460 nm and 250 mW for 1 h. **d** Azithromycin alone at 0.25 µg/mL. **e** Triple combination of AgNPs, blue light and the azithromycin. Signs of membrane damage and cell lysis were more pronounced in cells treated with a combination of three agents compared to cells treated with each agent alone. *Small arrows* indicate the location of the AgNPs and the sites of the damage

The antimicrobial efficiency of the AgNPs at 1/16 MIC, blue light for 1 h and azithromycin at 0.25 µg/mL against isolate N8 was visualized by TEM when each of them was used alone or in triple combination (Fig. 6a–e). Bacteria treated with AgNPs alone showed accumulation of the silver particles inside the cells concomitant with signs of membrane damage and lysis (Fig. 6b). Cell lysis was also observed when the bacteria were treated with either blue light or azithromycin alone (Fig. 6c, d). On the other hand, bacterial cell lysis was more pronounced following treatment with the three agents in combination, where the cells were severely affected (Fig. 6e).

## Discussion

Antibiotic resistance by isolates of *S. aureus* has become a global alarming problem that limits the availability of effective antimicrobial agents [22]. Antibiotic-misuse, the failure of some patients to comply with their treatment regimen, and the high capability of bacteria to mutate, are among the major factors contributing to the emergence of bacterial resistance. Antibiotic resistance leads to the failure of treatment of life-threatening bacterial infections and increases costs due to longer stay in healthcare settings [23, 24]. The use of non-conventional therapy to which bacteria are improbably to develop resistance, would be the best alternative. AgNPs are potential antimicrobials agents, which can be considered as an alternative to antibiotics for the treatment of infections caused by MDR bacteria [5]. AgNPs have been shown to possess strong and broad-spectrum antimicrobial activity due to a combined effect between their physical properties and the released free silver ions [25].

Methicillin-resistant *S. aureus* was used as a model in our study to assess the efficiency of combination of AgNPs, blue light, and anti-staphylococcal antibiotics. The isolates had been collected from different hospital units to guarantee the most possible representation of the Egyptian genotype population of *S. aureus*. We have previously found that members of CC8 are the prevailing MRSA clone in Egypt (Unpublished data, Master Thesis, Moussa et al. 2010). Infections caused by *S. aureus* are among the most frequent causes of both healthcare-associated and community-onset infections [26]. MRSA and coagulase-negative staphylococci are among the leading causes of nosocomial blood stream infections in the USA [27]. Staphylococci cause biofilm-associated infections by forming biofilms on damaged tissues, and indwelling vascular catheters [28–32].

Five antibiotics were selected from different conventional classes including beta-lactam (amoxicillin), macrolides (azithromycin and clarithromycin), oxazolidinones (linezolid), and glycopeptides (vancomycin). Based on the European Committee on Antimicrobial Susceptibility Testing (EUCAST) MIC breakpoint guideline [33], all isolates were found to be resistant to amoxicillin, azithromycin, clarithromycin, and linezolid, while they were susceptible to vancomycin (Table 2). With few exceptions, MRSA isolates are resistant to all beta-lactam antibiotics and commonly resistant to macrolides, with very rare resistance to glycopeptides antibiotics [33, 34]. Emergence of linezolid-resistance was previously reported in 0.05 % of *S. aureus* infections [35]. Antibiotics that are known to be ineffective against MRSA were used in this study to assess the possibility of enhancement of their antimicrobial activity by AgNPs and the blue light. This approach would be useful in “recycling” of these antibiotics that became useless against infections caused by MRSA to face the fast-growing drug-resistance with the slow development of new antimicrobial agents, and to preserve last resort antibiotics such as vancomycin.

Blue light has attracted increasing attention because of its intrinsic antimicrobial effect which does not involve the use of exogenous photosensitizers as in the photodynamic therapy (PDT), and the less damaging to mammalian cells than ultraviolet irradiation [13]. The biomedical applications of blue light at specific wavelengths and intensities against different pathogens have been reported earlier [36]. At 470 nm blue light was found to be efficient against MRSA strains associated with hospital-acquired and community-onset infections [37].

Double combinations of the AgNPs, at 1/2–1/128 of its MIC, with blue light were tested against selected MRSA isolates. The bactericidal activity of both agents was significantly enhanced (p < 0.001) when bacteria were treated with AgNPs with concurrent exposure to blue light for 1 h, compared to each of them alone (Fig. 1a–e). The combined therapy killed all bacteria after 8 h in all tested combinations while each of the silver compound and the blue light was less efficient in killing the organisms during the tested time. The mechanism of the antimicrobial effect of either AgNPs or blue light is still not fully understood. Several hypotheses have been suggested to explain such mechanisms. For example, it has been reported that AgNPs can damage bacterial cell membranes leading to structural changes, which render bacteria more permeable [38, 39]. AgNPs have unique optical, electrical, and thermal properties with high surface area to volume ratio resulting in the optimal possible interaction with bacterial surfaces leading to a higher antimicrobial activity [40]. The formation of free radicals by the silver nanoparticles is probably another mechanism that can lead to cell death [41, 42]. It has also been proposed that cationic silver is released from the nanoparticles when they are dissolved in water or when they penetrate into the cells [5]. Silver ions bind to the cellular membranes, proteins, and nucleic acids, causing structural changes and deformations of the bacterial cell [5]. They also deactivate many vital enzymes by interaction with thiol groups [31] and are involved in the generation of reactive oxygen species [32].

For blue light, the commonly accepted hypothesis is the production of highly cytotoxic reactive oxygen species (ROS) in a similar manner to PDT [43].

We have previously reported a similar synergistic interaction for a combination of AgNPs and blue light when both agents were tested against clinical isolates of *Pseudomonas aeruginosa* [44]. A possible mechanism of the observed synergy could be the transduction of the captured blue-light energy by blue light sensory proteins to the AgNPs resulting in the thermal destruction of the bacterial cells [45, 46].

Double combination of the AgNPs with five conventional antibiotics against the ten MRSA clinical isolates was investigated using checkerboard assay. Synergistic interactions were observed when the AgNPs were used with amoxicillin, azithromycin, clarithromycin or linezolid in 30–40 % of the combinations (Fig. 2). Other combinational activities were indifferent with no observed antagonistic interaction. Combination of the AgNPs with vancomycin, on the other hand, was indifferent against all isolates. Synergistic interactions between AgNPs and conventional antibiotics against different pathogens were previously reported. Ruden et al. [47] found synergistic interaction when AgNPs were combined with polymyxin B against gram-negative bacteria. Combination of AgNPs with ampicillin, chloramphenicol, and kanamycin against gram-positive and -negative pathogenic bacteria was also found to be synergistic [48]. Similarly, Smekalova et al. [49] observed synergistic effect when AgNPs were combined with penicillin G, gentamycin, and colistin against MDR bacteria.

Reversion of MRSA resistance to ineffective antibiotics by combination with AgNPs could be a novel strategy to combat infections caused by MDR pathogens. Biocompatible gold nanoparticles-amoxicillin complex was found to overcome the resistance of MRSA to the antibiotic [50]. The synergistic response of combination of AgNPs and ineffective antibiotics is probably due to an increase of the concentration of antibiotics at the site of bacterium–antibiotic interaction, and to facilitate binding of antibiotics to bacteria [51].

AgNPs combined with amoxicillin, azithromycin, clarithromycin or linezolid were tested against selected MRSA isolates with concurrent exposure to blue light for 1 h. The isolates were selected based on synergistic response, where they were most affected by the previous double combinations in the checkerboard assay. The antimicrobial activity of the three agents was significantly (p > 0.001) enhanced in the triple combinations compared to single- and double treatments with one or two of them. The bactericidal activity was more pronounced when azithromycin or clarithromycin was included in the triple therapy (Figs. 3, 4). The antimicrobial efficiency was also enhanced when linezolid (Fig. 5) or amoxicillin (data not shown) was included but with the same bactericidal effect in double and triple combinations.

The synergy observed in triple therapy might be explained by the combined mechanism of action of each agent alone, and the enhanced outcomes in their double combinations.

TEM images of isolate N8 that has been exposed to triple therapy (AgNPs, blue light and azithromycin) support the aforementioned suggestion that bacterial cell damage by the triple combination was more pronounced compared to the cells that were treated with each agent alone (Fig. 6a–e).

## Conclusions

This study suggests a new strategy to combat serious infections caused by MDR bacteria. The triple combination of AgNPs, blue light, and antibiotics is a promising therapy for infections caused by MRSA. The triple therapy may include antibiotics, which are proven to be ineffective against MRSA. This approach would be useful to face the fast-growing drug-resistance with the slow development of new antimicrobial agents, and to preserve last resort antibiotics such as vancomycin. The study can be taken further by exploring the application of the triple therapy in patients infected with MRSA and other MDR bacteria, taking into consideration the best conditions for optimizing their synergistic effects and decreasing the harmful side effect.

## Abbreviations

AgNPs: silver nano particles
AMX: amoxicillin
AZM: azithromycin
CFU/mL: colony forming units per milliliter
CLR: clarithromycin
CLSI: Clinical Laboratory Standard Institute
EUCAST: European Committee on Antimicrobial Susceptibility Testing
FIC: Fraction Inhibitory Index
LNZ: linezolid
MBC: minimum bactericidal concentration
MDR: multi-drug resistant
MHB: Mueller Hinton Broth
MIC: minimum inhibitory concentration
MLST: multiple locus sequence typing
MRSA: methicillin-resistant *Staphylococcus aureus*
mg/L: milligram per liter
mW: milliwatt
OXA: oxacillin
PDT: photodynamic therapy
PVP: polyvinylpyrrolidone
ROS: reactive oxygen species
TEM: transmission electron microscope
VAN: vancomycin
